# KIT V560D-Mutated Systemic Mastocytosis Associated With High-Risk Myelodysplastic Syndrome: A Unique Case of Systemic Mastocytosis–Associated Hematologic Neoplasm

**DOI:** 10.1155/crh/4360304

**Published:** 2024-11-30

**Authors:** Georgio Medawar, Krishna Sakalabaktula, Jenna Magri, Elizabeth Rinker, Praneeth Baratam

**Affiliations:** ^1^Department of Hematology-Oncology, Medical University of South Carolina, Charleston, South Carolina, USA; ^2^Department of Medicine, Rangaraya Medical College, YSR University of Health Sciences, Kakinada, Andhra Pradesh, India; ^3^Department of Pathology, Medical University of South Carolina, Charleston, South Carolina, USA

**Keywords:** avapritinib, midostaurin, myelodysplastic syndrome, outcomes, systemic mastocytosis

## Abstract

Systemic mastocytosis (SM) is a rare hematologic disorder characterized by clonal proliferation of mast cells in the bone marrow and/or other organs. SM-associated hematologic neoplasm (SM-AHN) is one of the advanced SM variants that usually confer a poor prognosis. We present a case of a 75-year-old female patient with SM-AHN, specifically myelodysplastic syndrome (MDS), that harbored a unique KIT mutation KIT V560D, not previously described in the literature in this setting. We describe the clinical course and the outcome with the use of avapritinib, midostaurin, and decitabine-cedazuridine.

**Trial Registration:** ClinicalTrials.gov identifier: NCT00782067

## 1. Introduction

Systemic mastocytosis (SM) is a rare hematologic disease characterized by an abnormal expansion and accumulation of pathologic mast cells (MCs) in multiple extracutaneous organs. The diagnosis can be established if the patient fulfills at least 1 major criterion and 1 minor criterion or 3 minor criteria (Table 1) [1–4]. The most common mutation in adult patients with SM is a gain-of-function KIT D816V type 3 tyrosine kinase receptor mutation, resulting in autonomous growth and expansion of neoplastic MCs. Other rare KIT mutations include V560G, V530I, K509I, D816Y, and D816H [1].

SM is further classified into indolent SM, smoldering SM, and advanced SM variants, including aggressive SM, mast cell leukemia (MCL), and SM with associated hematologic neoplasm (SM-AHN), previously known as SM with associated clonal hematologic non-MC lineage disease (SM-AHNMD) [2]. SM-AHN is a common presentation of mastocytosis, constituting approximately 30%–40% of the systemic mastocytosis cases [5]. In addition to KIT mutation, patients with SM-AHN often harbor an additional genetic defect correlating to a myeloid neoplasm, such as chronic myelomonocytic leukemia (CMML), myelodysplastic syndrome (MDS), acute myeloid leukemia (AML), myeloproliferative neoplasm (MPN), and chronic eosinophilic leukemia (CEL) [2, 6]. The diagnosis of the AHN on bone marrow biopsy can be difficult, as a prominent MC infiltration can make the associated hematologic malignancy subtle. Alternatively, the associated hematologic neoplasm may obscure MC infiltration, making accurate diagnosing difficult [5]. The mainstay treatment in indolent SM is management of chronic symptoms related to the release of MC mediators [1], whereas for more advanced disease, drugs aimed to reduce to MC burden are used to prevent end-organ damage [3]. The kinase inhibitors imatinib, midostaurin, and more recently, avapritinib have been approved by the Food and Drug Administration (FDA) for patients with advanced SM [1].

Herein, we report a unique case of a patient with SM-AHN with a KIT V560D mutation, in the setting of a high-risk cytogenetic and molecular MDS. We describe the diagnostic challenges we encountered, the clinical course, the treatment, and the outcome and we review the literature.

## 2. Patient Case

**Figure 1:**
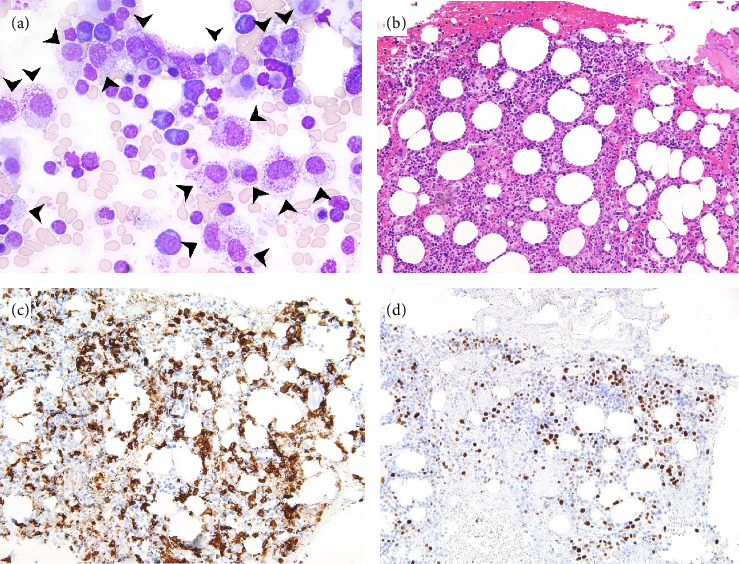
Histopathologic images of the first bone marrow biopsy. (a) Bone marrow aspirate showing numerous atypical, predominantly mature, mast cells with large and hypogranular forms (black arrowheads) (wright-giemsa stain; 600x). (b) Bone marrow core biopsy with approximately 40% cellularity showing increased mast cells (H&E, 200x). (c) Bone marrow core biopsy showing increased diffusely scattered mast cells positive for cytoplasmic CD117 immunohistochemical staining comprising approximately 10%–20% of the total cellularity (200x). (d) Bone marrow core biopsy showing strong positive nuclear p53 staining in scattered cells (200x).

A 75-year-old woman with a past medical history of hypertension, hyperlipidemia, hypothyroidism, osteoporosis, gastrointestinal stromal tumor (status post wedge partial gastrectomy in 2004), and early-stage gastric adenocarcinoma (status post subtotal gastrectomy and D2 lymphadenectomy in 2009) presented to the hospital in August 2023 with recurrent episodes of light-headedness, dizziness, near syncope, and syncope of 3 months duration. She reported associated facial flushing, nausea, rash, and vomiting. CBC with differential was unremarkable except for normocytic anemia of 10.5 g/dL. Further workup showed an elevated serum tryptase level of 425 ng/mL (reference range < 11.5 ng/mL). Skin biopsy showed superficial perivascular dermatitis and actinic skin damage but did not demonstrate a significant increase in MC number. Subsequently, a bone marrow biopsy was done and showed 40% cellularity with scattered MCs that constitute 10%–20% of the total cellularity. Bone marrow aspirate showed 27% atypical mature MC hyperplasia (large, hypogranular), dysmegakaryopoiesis, and a few ring sideroblasts (Figure 1). There was no significant clustering of MCs within the marrow and no evidence of immunophenotypic aberrancy, with CD-2, CD-25, and CD-30 negative in MCs. There were less than 5% blasts. Cytogenetics showed two distinct clonal populations: major clone (hyperdiploid with gain of *X*, 1p, deleted 1, deleted 3, 4, deleted 4, 8, 18, 19, and 21, abnormal 9 with material on 9p, two copies of an apparent abnormal 13 with additional material on 13p and deletion 13q, additional material on 21p, and a marker chromosome of uncertain origin), minor clone (with deletion of 5q and an abnormal 17 with 9q on 17p). Next-generation sequencing (NGS) identified mutations in TP53 P191del with variant allelic frequency (VAF) of 69%, KIT V560D (VAF 5%), and KMT2A T1092Yfs⁣^∗^ 51 (VAF 9%). The patient met 3 minor diagnostic criteria for SM (> 25% atypical bone marrow MCs, detection of KIT mutation, and serum tryptase persistently > 20 ng/mL). Also, despite not meeting the diagnostic morphologic criteria of MDS (low dysplastic burden in the bone marrow), the presence of the TP53 mutation with VAF of > 50%, KMT2A mutation, and the independent minor neoplastic clone with myeloid features raised the suspicion of an evolving concurrent high-risk MDS.

**Figure 2:**
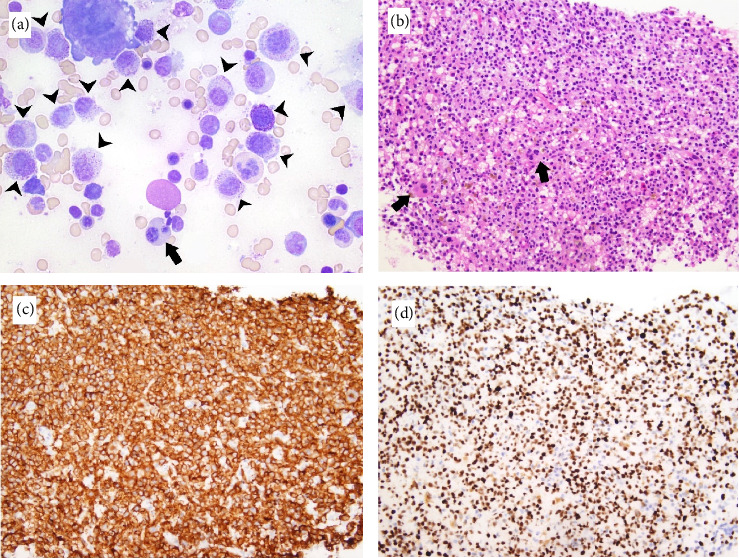
Histopathologic images of the second bone marrow biopsy. (a) Bone marrow aspirate showing numerous atypical, predominantly immature, mast cells with large and hypogranular forms (black arrowheads), and occasional dysplastic erythroid precursors, including a binucleated form (black arrow) (Wright–Giemsa stain; 600x). (b) Bone marrow core biopsy showing hypercellular (70%–80% cellularity) marrow with sheets of atypical mast cells and occasional dysplastic megakaryocytes (solid black arrows) (H&E, 200x). (c) Bone marrow core biopsy showing large clusters and sheets of mast cells, comprising approximately 70% of the total cellularity, that are positive for cytoplasmic CD117 immunohistochemical staining (200x). (d) Bone marrow core biopsy showing strong and diffuse nuclear p53 staining (200x).

The patient's tryptase level continued to rapidly increase, reaching 1276 ng/mL after 1 month. She started on avapritinib in early September 2023 at a dose of 200 mg once daily. Her antihistamine and MC stabilization medications (cetirizine, diphenhydramine, montelukast, and cromolyn) were continued at maximal dosage. She had a complete symptomatic resolution of symptoms with no recurrent episodic events for almost 3 weeks. However, symptoms relapsed afterward with 1-2 episodes per week, resulting in multiple hospitalizations. Her tryptase levels continued to increase, reaching 1901 ng/mL. A repeat bone marrow biopsy was done in November 2023 and showed progressive expansion of the MC clone (40% of aspirate and 70% of core). The MCs were atypical immature: large and hypogranular with occasional bilobed forms. They formed large clusters and sheets throughout the marrow exhibiting strong and diffuse nuclear p53 expression and cytoplasmic CD117 positivity by immunohistochemistry (IHC) (Figure 2). This suggested progression of systemic mastocytosis with features compatible with MC leukemia, aleukemic variant. The background was still showing trilineage hematopoiesis with only mild dysplasia. Blast cell percentage was 3%. Cytogenetics redemonstrated one complex abnormal clone associated with systemic mastocytosis/MCL and a second independent myeloid neoplasm with high-risk features, including loss of 5q and biallelic loss of 17p (TP53). NGS showed the same mutational profile with increased VAF in TP53 mutation (up to 72%), in KIT V560D mutation (up to 11%), and in KMT2A (up to 29%).

Avapritinib was continued and decitabine-cedazuridine (Inqovi 35–100 mg) was started in mid-November 2023, as a way to decrease the MDS burden in the bone marrow. A 5-day cycle of inqovi was completed. However, the patient was admitted to the hospital 2 weeks later for pleural effusions and volume overload, after which avapritinib was discontinued. Midostaurin 100 mg BID was started in mid-December 2023 while awaiting prior authorization for ripretinib. Cycle 2 of inqovi was delayed due to significant fatigue and deconditioning. Unfortunately, the patient was hospitalized a few days later for altered mental status, persistent fevers, and acute respiratory failure. She was found to have an acute subdural hematoma (with platelet count 122 K/cumm, Hemoglobin 8.2 g/dL, INR 1.09, aPTT 36 s (ref 25–36)), large pleural effusions, and type II NSTEMI. Due to the aggressive nature of her disease and the multiple acute medical problems, the family decided to move forward with comfort care and the patient passed away on January 1, 2024.

## 3. Discussion

### 3.1. Challenging Diagnosis

The initial SM diagnosis was considered based on 3 minor criteria listed (> 25% atypical bone marrow MCs, detection of KIT mutation, and serum tryptase persistently > 20 ng/mL), despite having another evolving small myeloid clone and mild bone marrow dysplasia. The WHO classification does not consider the tryptase level as a minor criterion if there is an associated myeloid neoplasm [2, 6]. However, in our case, due to worsening symptoms and rapidly rising tryptase (reaching > 1000 ng/mL) with relatively stable MDS (stable cytopenias, no increase in peripheral blasts, and no new B-symptoms), SM-AHN diagnosis was established. The small MDS component was morphologically benign but cytogenetically and molecularly aggressive, making it very high risk based on the International Prognostic Scoring System for Myelodysplastic Syndromes-Molecular (IPSS-M score = 1.57). Also, although the initial bone marrow aspirate had 27% atypical MCs, the predominant cells lacked convincing features of immaturity (promastocytes, metachromatic blast-like forms, and multinucleated or highly pleomorphic cells), making MCL, which requires meeting diagnostic criteria for SM with > 20% atypical immature MCs, less likely [7].

The patient had a KIT V560D activating mutation, which, to our knowledge, is the first reported mutation variant associated with SM. KIT V560D, in contrast to KIT D816D which occurs at exon 17, is an activating mutation at exon 11 that affects the juxta membrane domain on the KIT receptor which is most commonly associated with GIST [8]. Interestingly, our patient had a history of surgically resected GIST, which was in remission, and later developed SM with no evidence suggestive of germline mutation in KIT V560D (low VAF). We did not have the information on the molecular panel at the time of her GIST diagnosis, which limited us from establishing a temporal relation to the KIT V560D mutation, if present initially.

Unfortunately, MCL transformation, aleukemic variant (with no circulating MCs) occurred 3 months after the initial diagnosis of SM. The diagnosis was based on bone marrow biopsy morphology showing 70% atypical immature MCs forming large clusters and sheets throughout the marrow and bone marrow aspirate showing 40% atypical immature cells that include bilobated promastocytes.

### 3.2. Challenging Treatment

Treatment of adult SM varies individually, and in the case of SM-AHN subtype, the treatment varies depending on the component that causes a major burden on the patient [1]. The integration of clinical, histological, and molecular data helps in assessing which component of the disease warrants immediate treatment [1]. The SM component in our patient was causing a high symptomatic burden despite optimal medical therapy, which prompted us to treat it as aggressive SM. KIT-directed treatment options include imatinib, avapritinib, and midostaurin. Other options include cladribine, clinical trials, and allogeneic stem cell transplant [9].

- Imatinib is a type 2 kinase inhibitor that demonstrates in vitro efficacy against wildtype KIT and certain transmembrane (F522C) and juxtamembrane (V560G) KIT mutants but not the common kinase (D816V) domain mutants found in SM [1, 10]. In 2006, the FDA approved imatinib for adult patients with aggressive SM (ASM) without the D816V KIT mutation or with unknown or unavailable KIT mutational based on clinical data from single case reports and a small series of patients with mastocytosis treated with this drug [11].
- Midostaurin is a type 1 multikinase inhibitor of KIT D860V, WT KIT, PDGFR *α*/*β*, VGFR2, and FLT3. It also inhibits the IgE-dependent release of histamine and the growth of neoplastic MC with various KIT mutations [10]. It was FDA approved as a treatment for AdvSM in April 2017 based on the phase 2, open-label trial in which 116 patients were enrolled of which 89 were primary efficacy population (16 with ASM, 57 with SM-AMN, and 16 with MCL) [12, 13]. The ORR was 60% (45% major response and 15% partial response) irrespective of the type of AHN present, D860V status, or prior therapy. After a median follow-up of 26, the median duration of response was not reached in ASM or MCL patients and was 12.7 months in SM-AMN patients. The median overall survival (OS) for ASM, SM-AMN, and MCL patients was not reached, 20.7 months and 9.4 months, respectively. The progression-free survival (PFS) for the three groups of patients was 28.7, 11, and 11.3 months, respectively. Significant reduction in serum tryptase levels (−58%), bone marrow MC burden (−59%), and spleen size were observed [9]. The most common nonhematological drug side effects (all grades/grades 3–4) were nausea (79%/6%), vomiting (66%/6%), diarrhea (54%/3%), and fatigue (28%/9%). Hematological side effects (all grades) are neutropenia, anemia, and thrombocytopenia which occurred in 48%, 63%, and 52% of the patients [13].
- Avapritinib is a type 1 highly selective multikinase inhibitor of KIT D860V and PDGFR*α* and is 10-fold more potent compared to the midostaurin in vivo in inhibiting KIT D860V [10]. Based on the EXPLORER and PATHFINDER trials, the FDA has approved avapritinib as the first line of treatment for adult patients with AdvSM that also includes ASM, SM-AHN, and MCL in June 2021 [14]. In Phase 1 EXPLORER trial among 86 patients of which 69 are Adv-SM, it was associated with an estimated 24-month OS of 76% in all patients with AdvSM (100%, 67%, and 92% for the ASM, SM-AHN, and MCL subtypes, respectively). The overall estimated PFS was 63% at 24 months. The overall response rate (ORR) was 75%. Greater than 50% reduction in BM MC in 92% of the patients and serum tryptase level was observed in 99% of the patients. The KIT D816V VAF was reduced from the baseline by ≥ 50% and became undetectable in 80% and 30% of the patients, and the estimated PFS rates at 12 and 24 months were 84% and 63%, respectively [15]. In phase 2 PATHFINDER trial interim analysis, the ORR evaluated among 32 patients with AdvSM was 75%. Similarly, a 50% reduction of bone marrow MC count was seen in 88% of patients. The estimated PFS rate at 12 months was 79%. The OS rate at 12 months was 86% [16]. Adverse effect profile reported in the EXPLORER trial included periorbital edema (69%), anemia (55%), diarrhea (45%), thrombocytopenia (44%), nausea (44%), intracranial bleeding (13%), and cognitive dysfunction (30%). The side-effect profile in the PATHFINDER trial was similar but with a lower percentage [15, 16].

For both midostaurin and avapritinib, none of the conducted trials included a KIT V560D mutation, which is not previously described in SM, SM-AHN, or MCL. Imatinib was reported by Heinrich et al. to be active in vitro against an isolated KIT V560D clone with IC50 of around 100 nmol/L [17]. Similarly, in another study, avapritinib was reported to have in vitro activity with an average IC50 of 60 nmol [18]. Based on these preclinical data, we proceeded with avapritinib at the approved dose for SM. Despite transient clinical improvement that lasted 3 weeks, symptoms relapsed, and a repeat bone marrow showed transformation to MCL. Avapritinib was discontinued due to volume overload and was switched to midostaurin (while awaiting ripretinib approval), and the patient was started on MDS-directed therapy with decitabine-cedazuridine. Due to her age, comorbidities, and performance status, allogeneic stem cell transplantation referral was deferred. Other treatments that were considered for the patient were off-label use of ripretinib, which was FDA approved in 2020 for the treatment of advanced GIST, and is highly potent against KIT V560D (with IC50 of 4 ± 1.8 nmol), regorafenib, which is also highly potent against KIT V560D (with IC50 of 4.7 nmol), and cladribine [19, 20]. Unfortunately, the patient's clinical condition rapidly deteriorated, precluding further treatments.

### 3.3. Outcome

As mentioned above, SM-AHN and MCL had worse PFS and OS as compared with ASM in the trial (midostaurin) and the Explorer trial (avapritinib). In a 2009 study by Lim et al., the reported median OS for SM-AHN was 24 months (13 months specifically in SM-MDS). Overall, the prognosis of MCL is significantly worse than other subtypes of SM, with an OS ranging from 0.5 to 2.6 years, according to some studies. Furthermore, patients with MCL-AHN had a more inferior OS (1.6 years) [7]. Our patient survived for 4 months after her diagnosis and her disease was in relapse at the time of death. Her immediate cause of death was intracranial bleeding despite acceptable platelet count and normal coagulation studies. Multiple factors might have contributed to that, including the use of avapritinib (13% intracranial bleeding events in the EXPLORER trial) and/or the MC disease itself. The risk of bleeding increases in MC disorders due to, at least in part, degranulation products such as heparin, histamine, and tryptase and by fibrinolysis [21].

## 4. Conclusion

In this very rare case of KIT V560D-mutated SM-AHN with high-risk MDS, the disease was refractory to avapritinib, midostaurin, and decitabine-cedazuridine. This poor outcome may be elucidated by KIT V560D insensitivity to avapritinib/midostaurin, the high TP53 mutational burden, and/or the rapid transformation to MCL. Based on this report, KIT V560D could be potentially added to the list of rare KIT mutations associated with SM.

## Figures and Tables

**Table 1:** Proposed refined major and minor SM criteria [4].

Major criterion	Multifocal dense infiltrates of mast cells (≥ 15 mast cells in aggregates) in bone marrow biopsies and/or in sections of other extracutaneous organ(s)

Minor criteria:	a. ≥ 25% of all mast cells are atypical cells (Type I or Type II) on bone marrow smears or are spindle shaped in mast cell infiltrates detected in sections of bone marrow or other extracutaneous organs^a^
	b. KIT-activating KIT point mutation(s) at codon 816 or in other critical regions of KITb in bone marrow or another extracutaneous organ
	c. Mast cells in bone marrow, blood, or another extracutaneous organ express one or more of: CD2 and/or CD25 and/or CD30c
	d. Baseline serum tryptase concentration > 20 ng/mL (in the case of an unrelated myeloid neoplasm, an elevated tryptase does not count as an SM criterion). In the case of a known H*α*T, the tryptase level should be adjustedd
	If at least 1 major and 1 minor or 3 minor criteria are fulfilled ⟶ the diagnosis is SM

Abbreviations: H*α*T, hereditary alpha-tryptasemia; SM, systemic mastocytosis.

^a^In tissue sections, an abnormal mast cell morphology counts in both a compact infiltrate and a diffuse (or mixed diffuse + compact) mast cell infiltrate. However, the spindle-shaped form does not count as an SM criterion when mast cells are lining vascular cells, fat cells, nerve cells, or the endosteal-lining cell layer. In the bone marrow smear, an atypical morphology of mast cells does not count as SM criterion when mast cells are in or adjacent to bone marrow particles. Morphologic criteria of atypical mast cells have been described previously.

^b^Any type of KIT mutation counts as minor SM criterion when published solid evidence for its transforming behavior is available.

^c^All 3 markers fulfill this minor SM criterion when expression in mast cells can be confirmed by either flow cytometry or by immunohistochemistry or by both techniques.

^d^Although the optimal way of adjustment may still need to be defined; one way is to divide the basal tryptase level by 1 plus the extra copy numbers of the alpha tryptase gene. Example, when the tryptase level is 30 and 2 extra copies of the alpha tryptase gene are found in a patient with H*α*T, the H*α*T-corrected tryptase level is 10 (30/3 = 10) and thus is not a minor SM criterion.

## Data Availability

The data that support the findings of this study are available from the corresponding author upon reasonable request.
